# Exploring User Needs and Preferences for Mobile Apps for Sleep Disturbance: Mixed Methods Study

**DOI:** 10.2196/13895

**Published:** 2019-05-24

**Authors:** Melissa Aji, Christopher Gordon, Dorian Peters, Delwyn Bartlett, Rafael A Calvo, Khushnood Naqshbandi, Nick Glozier

**Affiliations:** 1 Brain and Mind Centre Central Clinical School, Sydney Medical School Faculty of Medicine and Health, University of Sydney Camperdown NSW Australia; 2 CRC for Alertness, Safety and Productivity Melbourne, Victoria Australia; 3 Centre for Sleep and Chronobiology, Woolcock Institute of Medical Research Glebe Australia; 4 Susan Wakil School of Nursing and Midwifery, Faculty of Medicine and Health, The University of Sydney Sydney Australia; 5 School of Electrical and Information Engineering, University of Sydney Sydney Australia; 6 Central Clinical School, Sydney Medical School, Faculty of Medicine and Health, The University of Sydney Sydney Australia; 7 Dyson School of Design Engineering, Imperial College London London United Kingdom

**Keywords:** mobile apps, mHealth, sleep

## Abstract

**Background:**

Mobile health (mHealth) apps demonstrate promise for improving sleep at scale. End-user engagement is a prerequisite for sustained use and effectiveness.

**Objective:**

We assessed the needs and preferences of those with poor sleep and insomnia to inform the development of an engaging sleep app.

**Methods:**

We triangulated results from qualitative (focus groups and app reviews) and quantitative (online survey) approaches. A total of 2 focus groups were conducted (N=9). An online survey tested themes identified from the focus groups against a larger population (N=167). In addition, we analyzed 434 user reviews of 6 mobile apps available on app stores.

**Results:**

Common focus group themes included the need to account for diverse sleep phenotypes with an adaptive and tailored program, key app features (alarms and sleep diaries), the complex yet condescending nature of existing resources, providing rationale for information requested, and cost as a motivator. Most survey participants (156/167, 93%) would try an evidence-based sleep app. The most important app features reported were sleep diaries (148/167, 88%), sharing sleep data with a doctor (116/167, 70%), and lifestyle tracking (107/167, 64%). App reviews highlighted the alarm as the most salient app feature (43/122, 35%) and data synchronization with a wearable device (WD) as the most commonly mentioned functionality (40/135, 30%).

**Conclusions:**

This co-design process involving end users through 3 methods consistently highlighted sleep tracking (through a diary and WD), alarms, and personalization as vital for engagement, although their implementation was commonly criticized in review. Engagement is negatively affected by poorly designed features, bugs, and didactic information which must be addressed. Other needs depend upon the type of user, for example, those with severe insomnia.

## Introduction

Sleep disturbances are widespread, impacting 33% to 45% of adults [1]. The most common sleep disorder is insomnia which is underrecognized and undertreated [2]. The most limiting treating factor is a shortage of treatment resources including trained clinicians [3]. In response, there has been increasing interest in harnessing mobile technology for sleep health. The mobile app marketplace has expanded rapidly with approximately 325,000 mobile health (mHealth) apps now available across major app stores [4].

Existing mHealth sleep apps offer a broad range of functions including sleep tracking, education, alarm clocks, and sound recording during sleep. A recent review examining mobile phone interventions for sleep suggested that mobile apps are effective in improving symptoms and sleep quality [5]. However, engagement is often poor: Approximately half of respondents who download health apps no longer use these owing to poor user experience [6]. Moreover, users are likely to abandon an app that fails to provide immediate engagement [7].

Critical to developing an engaging tool is an understanding of the needs, contexts, and preferences of end users, including functional (ie, what people need and expect an app to do) and nonfunctional requirements (ie, use of relevant and appealing language and visuals). We also need to understand the context of technology use (eg, will users sleep with their phone by the bed and use other sleep aids with the app?) and the characteristics of prototypical users (personas). Therefore, in developing a new sleep app [7], we undertook a mixed methods user-centric approach to determine the needs and preferences for sleep apps in those with poor sleep or insomnia.

## Methods

### Design

We triangulated data [8] from 3 end user–centric methods: focus groups, an online survey, and an analysis of app reviews to give a holistic understanding by harnessing both qualitative research to provide rich insights and quantitative research to examine trends in a larger population. This counterbalances inherent biases in each method with strengths inherent to the other methods.

We addressed poor quality sleep as a continuum from disturbed sleep to insomnia for the focus groups and the survey. Both sample participants were aged ≥18 years and screened for poor sleep and insomnia using the Pittsburgh Sleep Quality Index (PSQI) and Insomnia Severity Index (ISI), respectively.

Ethical approval for the focus groups and survey was obtained from the Sydney Local Health District Ethics Committee (X17-0177). Before participation, focus group participants provided written consent and survey participants provided digital consent.

#### Pittsburgh Sleep Quality Index

The PSQI [9] assesses subjective sleep quality over the previous month. It comprises 7 components added to yield one *global* score (range 0-21). Higher scores represent poorer subjective sleep quality. A score of above 5 indicates poor sleep quality.

#### Insomnia Severity Index

The ISI [10,11] is a 7-item questionnaire (score range 0-28) assessing insomnia symptom severity over the past 2 weeks with higher scores indicating greater insomnia symptom severity and moderate-severe insomnia defined as >15.

### Focus Groups

#### Participants and Recruitment

Invitations were sent to participants on the Woolcock Institute of Medical Research database. Eligible participants were fluent in English and assessed for Insomnia Disorder (Diagnostic and Statistical Manual of Mental Disorders) by (1) a telephone screening checklist with a study coordinator, (2) score ISI > 15, and (3) PSQI ≥5 plus component 5 (sleep disturbance) total score of 1 or 2.

#### Procedure

In total, 2 activity-based workshops (1.5 hours each) were conducted by a user experience specialist (DP) using semistructured topic guides and activities. Workshops explored participants (1) experience of poor sleep, (2) experience using technology to help with sleep, and (3) ideas and preferences for a sleep app. Activities including collaborative ideation and paper prototyping explored several user experience questions drawn from literature on participatory design [12-14]. Participants generated ideas for desirable functionalities and app characteristics and provided feedback on draft screen designs for a prototype app designed by the research team. The data collected included participant-generated artifacts (ie, filled-in advertisement templates and screen design sketches), collections of feature ideas, field notes, and audio recordings and workshop transcripts.

#### Data Analysis

Participatory workshops were recorded and transcribed verbatim using NVivo (QSR International Pty Ltd Version 11.4.3, 2017). These transcripts were analyzed using thematic analysis [15], consistent with methods of analysis of generative participatory data [12].

Thematic analysis focused on categorizing data to inform development (1) features, content, and characteristics of an ideal app and (2) experience of sleep problems that could be addressed via technology. Initial coding (DP and KN) involved attaching labels to text segments to identify themes related to the research questions. Analysis progressed iteratively with independent rereading of transcripts and reexamining themes against the raw data to further refine the themes and identify subthemes. Discrepancies in coding were regularly discussed and resolved. A thematic schema was developed using FreeMind software 0.9.0.

### Survey

#### Participants and Recruitment

An anonymous online survey was conducted between May and July 2018. Facebook and Instagram advertisements invited participants to participate in a 20-min survey to help design a mobile app for sleep. Individuals were eligible if online screening indicated positive for poor sleep (PSQI ≥5) or insomnia symptoms (ISI >15) and provided informed online consent. Upon completion, participants could enter a draw to win a gift card (valued at AUD $50). All participants could receive a summary of the findings by including their email address. Responses were stored on a password-protected database on a secure server.

#### Survey Content

The survey assessed (1) demographics, (2) mobile phone usage, (3) wearable device (WD) usage, (4) sleep environment, (5) preferences for a sleep app, and (6) previous health app and website usage.

In total, there were 34 questions, with an open-ended question about likes and dislikes of existing digital health technologies (Multimedia Appendix 1 for survey).

#### Data Analysis

Descriptive statistics were performed for all items. Chi-square tests compared proportions of sleep app preferences across groups defined by insomnia severity (ISI <17 vs ISI ≥17) and WD ownership (WD vs no WD). Analyses were performed using SPSS version 24.

#### App Reviews

Existing studies exploring user experience of online interventions often focus on perspectives of treatment completers or active engagers [16,17], limiting the sample to satisfied users who persist with the program. On the contrary, app reviews garner perspectives from a larger and more diverse sample. They are often written by users with varying satisfaction including those who are extremely dissatisfied and have given up on the app.

#### Sample

In February 2018, an electronic search was conducted on Google Play and the App Store (United States) using the following search terms: *sleep*, *sleep health*, and *sleep therapy*. Apps were included if they (1) were consumer targeted, (2) targeted adults, (3) included the term *sleep* in the app title or description, (4) included personalized sleep feedback, (5) allowed for sleep tracking with a WD, and (6) had an English interface.

Apps were excluded if they were developed with the sole function of sleep tracking, relaxation sounds, hypnosis, meditation, recording of snoring and sleep talking, and/or alarm clocks. Apps were also excluded if they (1) required health care provider guidance, (2) were only accessible via an employer, (3) had fewer than 10 reviews, or (4) were a companion app for a sleep measurement device.

#### Analysis of User Reviews

Qualitative content analysis was used to code the app user reviews [18]. A pilot coding scheme of major and subcategories was iteratively developed from a random sample of 165 reviews from the 6 apps (listed in the results). This coding scheme also adapted themes identified in previous studies reviewing app user feedback [19,20] including the Components of User Experience model [21]. In total, 2 coders (MA and KN) agreed on the final coding scheme (Multimedia Appendix 2).

The team independently coded user reviews line-by-line and classified as *praise* or *critique*. Coding disputes were resolved, and any new codes detected from analysis were discussed and incorporated into the coding scheme.

## Results

### Focus Groups

#### Sample

A total of 9 participants (6 males and 3 females, aged 21 to 70 years) were recruited.

#### Workshops

**Figure 1:**
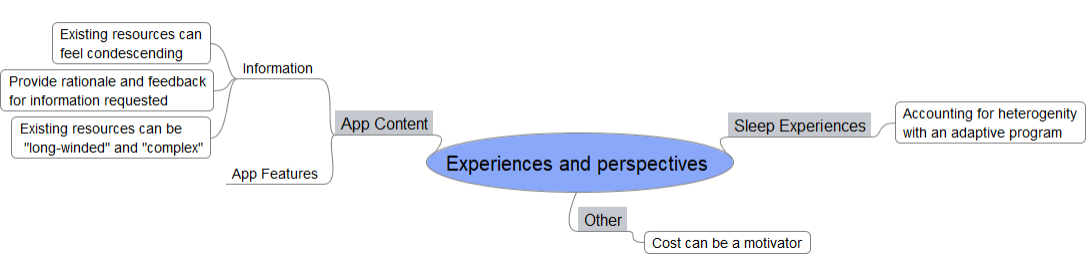
Thematic schema.

The participant’s needs and preferences for an engaging sleep app formed overarching themes of *sleep experiences*, *app content,* and *other* (Figure 1). A complete list is provided in Multimedia Appendix 3.

#### Main Themes and Insights

##### Sleep Experiences

###### Accounting for Heterogeneous Experiences With an Adaptive Program

Participants’ experience of sleep difficulty was diverse and included frequent night awakenings, trouble falling asleep, nightmares, snoring, partner disturbance, daytime stress and sleepiness, anxiety over not sleeping, and lack of a sleep rhythm. As such an ideal sleep program must be both customizable and adaptive to remain relevant to this wider population. Addressing the user’s specific sleep problems while catering for different lifestyles and schedules is crucial. A male participant’s statement illustrates both:

I find I’m so erratic with this...so a program that says, “Right, go to bed now”...sometimes I might go to sleep in a few minutes and have, not a good sleep, but doze. Other times, I can be as tired as possible, and I just can’t sleep...So, you know, “go to sleep for five hours”, and five hours later I’m still there looking at the ceiling. So if that didn’t work, “let’s try this approach”.

P3, FG2

##### App Content

###### App Features

Participants generated feature ideas including:

- Alarms for waking up and staying awake when tired, challenging alarm turn-off sequences to prevent oversleeping and a selection of alarm sounds
- Audio features (meditation, podcasts, and relaxing sounds)
- Encouragement (signs of progress and rewards)
- Frequently Asked Questions were preferred to chatbots as the latter requires keyboard input
- Recommendations for helpful apps and websites
- Social features, in particular, the ability to share information among a community of poor sleepers. There was interest in tips from others who have successfully completed the program.

Unsurprisingly, there was a strong interest in sleep tracking and data visualization. As one participant [P3, FG2] stated, “Graphs appeal to me...Graphs, I like graphs.” Participants expressed interest in objective data (eg, “How much you sleep per night, and how much is REM, and how much is deep sleep”[P3, FG1]) but also stressed the importance of subjective measures such as sleep *quality* or *satisfaction* explaining that duration alone can be misleading:

It says how many hours of sleep do you get a night, well, nowhere in there does it ask for the quality of sleep...If it’s four, there are four hours, but it might be worth one out of ten.

P2, FG2

Tracking daily behaviors such as exercise or usage of treatments, for example, sleeping pills against sleep was important:

I think a graph that can be potentially smart enough to start linking some of these—you might try a tag, you know, “took magnesium”,...and then you can graph your sleep, your quality of sleep against those tags...I like the tags.

P3, FG2

##### Information

###### Existing Resources Can Feel Condescending

Some participants found certain online sleep programs presented overly basic content and quizzes:

One of the really aggravating things about the program was...you had to complete this course in the week and they gave you these little things to do. And you were treated as an absolute moron, all the way through this thing. It was, you know, “now, if you exercise, you know, an hour before going to sleep, you get your adrenaline running and everything, so it’s bad to exercise an hour before going to sleep”. And so they told you all of these things, and then at the end of it, you had to do this quiz. Now, “Jenny has just exercised an hour before going to sleep. Is this good or bad for her?” And so you had to fill out these things, and you’re just thinking, really? Do I really have to do this?...and you were just treated as an absolute moron. “Oh, you’ve got a hundred percent, well done.” And you’re just thinking, oh, my God, I’ve paid two hundred dollars to do this. And it was just really demeaning.

P4, FG1

###### Existing Resources Can Be Long-Winded and Complex

Several participants expressed frustration with existing online content and exercises for sleep being *too complicated*, *overwhelming*, *tedious*, and *long-winded*. One male participant explains:

But there’s so much stuff. And all these suggestions and all these relaxation exercises and so much stuff, I just, I can’t go through all this. It’s just driving me nuts. They’re just too complicated.

P2, FG2

This reflects the prevailing tension between the need for resources that are neither oversimplified and condescending nor too complex and overwhelming.

###### Provide Rationale and Feedback for Information Requested

Participants expressed frustration in filling out lengthy questionnaires without a clear rationale or immediate feedback on the data collected. A female participant explained:

Can I just say, with the questionnaires, the burden of answering so many questionnaires at times can be really—and I know that you’ve got to do this...but with an app, if it’s explained at the outset, “we’re going to ask you a lot of questions, and we apologise...for being such a difficult thing; but we’re going to give you feedback straightaway on what we can see so far, or what the issues that might actually be identifiable at the outset”. And if you actually had that right from the beginning...it would be much more intuitive, much more easy to use, and you’d actually feel benefit right from the start.

P3, FG1

##### Other

###### Cost Can Be a Motivator

Making a sleep app free may undermine long-term commitment. The female participant [P4, FG1] previously quoted as expressing frustration over the condescending program explained that she completed the program, but only as she had already invested money in it:

I just didn’t think it was two hundred dollars well spent. And really the only thing they told me was “limit your sleep”, and I didn't think that it worked for me.

Participant

But you made it to the end, so I’m curious, what do you reckon was the most motivating thing that got you to keep going even though you were...

Facilitator

The two hundred dollars...It’s two hundred dollars and I’m a desperate woman...

Participant

### Survey

#### Response Rates

The survey received 546 hits, with 186 people completing screening questionnaires. Of these, 19 were ineligible on the ISI and PSQI leaving 167 participants.

#### Demographics

Participants were aged between 18 and 79 years (mean 38.8, SD 11.9) and predominately female (88%; Table 1).

#### Technology Usage

Most participants (162/167, 97%) reported using a mobile phone daily (Table 2). A large proportion of participants reported almost always keeping their phone by their bed at night (142/167, 85%) and using their phone as an alarm clock (113/167, 68%). Approximately one-third (54/167, 32%) reported using a WD, with Fitbit (48%) as the most popular. The 3 most frequently used features of WD’s were tracking for steps (48/54, 88%), heart rate (39/54, 72%), and sleep (35/54, 65%). Over half (94/167, 56%) reported using a health-related app. Among the 56% (93/167) using mobile apps for sleep, the most frequently used were meditation apps (45/93, 48%).

No differences were found in sociodemographic, mobile phone or WD use between those with high or low levels of insomnia symptom severity nor were there any sociodemographic differences identified between those who use a WD (all *P*>.05).

**Table 1:** Sociodemographic characteristics of respondents (n=167).

Variable		n (%)
**Gender**		
	Female	147 (88.0)
	Male	18 (10.8)
	Prefer not to say	2 (1.2)
**Age (years)**		
	18-30	48 (28.7)
	31-40	55 (32.9)
	41-50	33 (19.7)
	51+	31 (18.6)
**Education**		
	Secondary school	46 (27.5)
	Diploma	25 (15.0)
	Trade certificate	29 (17.4)
	Bachelor’s degree	45 (26.9)
	Postgraduate degree	22 (13.2)
**Employment^a^**		
	Full-time	53 (31.7)
	Part-time	58 (34.7)
	Student	17 (10.2)
	Unemployed	20 (12.0)
	Retired	11 (6.6)
	Other	16 (9.6)

^a^Percentages do not add up to 100% as respondents were allowed multiple responses.

**Table 2:** Mobile phone, wearable device, and app usage (N=167).

Variable			n (%)
**Mobile phone brand**			
	Samsung		73 (43.7)
	Apple		70 (41.9)
	Other		24 (14.4)
**Wearable device**			
	**Usage**		
		Yes	54 (32.3)
		No	113 (67.7)
	**Brand**		
		Fitbit	26 (48.1)
		Apple	10 (18.5)
		Other	18 (33.4)
**Purpose^a^**			
	Fitness		45 (83.3)
	Health		36 (66.7)
	Communication		23 (42.6)
**Features used^a^**			
	Step tracking		48 (88.9)
	Heart rate monitoring		39 (72.2)
	Sleep tracking		35 (64.8)
**Health app usage**			
	Yes		94 (56.2)
	No		73 (43.7)

^a^Percentages do not add up to 100% as respondents were allowed multiple responses.

#### Sleep App Preferences

In total, 93% (156/167) of individuals indicated willingness to try an app proven to improve sleep. A sleep diary and tracking (148/167, 88%) the ability to share sleep data with their doctor (116/167, 70%), offline functionality (113/167, 68%), and tracking of lifestyle factors (107/167, 64%) were listed (Table 3). Only those participants with increased insomnia severity wanted to share sleep data with their doctor.

### App Reviews

#### Descriptive Analysis

**Figure 2:**
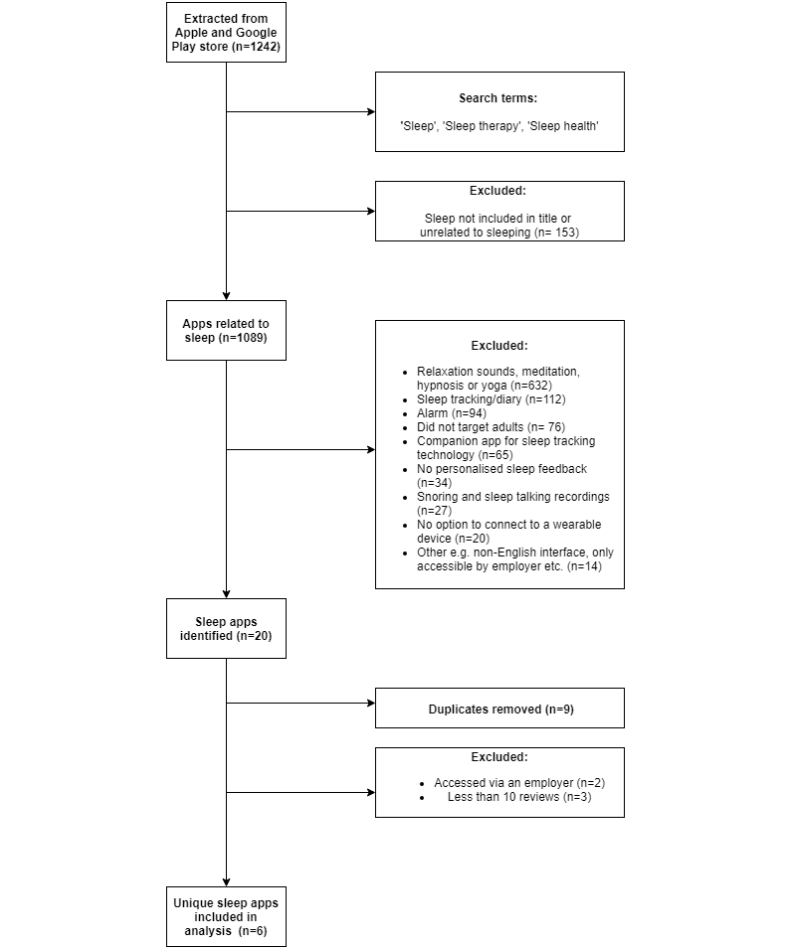
Preferred Reporting Items for Systematic Reviews and Meta-Analyses flow diagram of selected apps.

The initial search of all stores yielded 1242 apps, of which 1089 were sleep related (Figure 2), and 20 met the inclusion criteria. Apps were excluded if they were duplicates, had restrictive access, or <10 reviews. In total, 6 apps remained (Lark, Pillow, Sleep as Android, SleepHealth, Sleepio, and Sleeprate) and their reviews were analyzed (Multimedia Appendix 4 for app content). A total of 2 were in both in Google Play and the App Store, with the others found solely from either the App Store (3/6) or Google Play (1/6). Reviews for each app ranged from 11 and 246,240 (mean 62,327). The average ratings ranged from 2.7 to 4.5 out of 5 stars (mean 3.7). Of the 6 apps, 5 were free, but 4 offered a paid upgrade or paid in-app features. One app provided mobile access to a paid sleep web program.

Most were designed for commercial purposes, except for SleepHealth, which is restricted for research. The apps varied in the number of functions; all included a sleep diary and graphical feedback. All apps provided personalized advice based on sleep data, an accelerometer-derived sleep tracking (5/6), data relating to sleep stages (4/6), and sleep hygiene education (4/6).

**Table 3:** Preferences in sleep app features (abridged; N=167).

Features			n (%)	High insomnia (Insomnia Severity Index 17+)	Low insomnia	*P* value
**Importance of...**						
	**Sleep diary and tracking**					.32
		Little to no importance	19 (11.4)	9 (5)	10 (6)	
		Important	148 (88.6)	88 (53)	60 (36)	
	**Tracking of diet, exercise, and other lifestyle factors**					.11
		Little to no importance	60 (35.9)	30 (18)	30 (18)	
		Important	107 (64.1)	67 (40)	40 (24)	
	**Sharing or comparing sleep data with friends or other users**					.45
		Little to no importance	138 (82.6)	82 (19)	56 (34)	
		Important	29 (17.4)	15 (9)	17 (8)	
	**Sharing sleep data with your doctor**					<.001
		Little to no importance	51 (30.5)	20 (12)	31 (19)	
		Important	116 (69.5)	77 (46)	39 (23)	
	**Linking to a wearable device (eg, Fitbit, Jawbone, and ActiWatch)**					.58
		Little to no importance	84 (50.3)	47 (28)	37 (22)	
		Important	83 (49.7)	50 (30)	33(20)	
	**Being usable offline (without an internet connection)**					.14
		Little to no importance	54 (32.3)	27 (16)	27 (16)	
		Important	113 (67.7)	70 (42)	43 (36)	
	**Not requiring the phone to be by the bed**					.49
		Little to no importance	110 (65.9)	66 (40)	44 (26)	
		Important	57 (34.1)	31 (19)	26 (16)	

#### Sample

For each app, the 100 most recent reviews were included except if there were fewer than 100 reviews (3 out of 6 apps), resulting in 434 reviews. Reviews were excluded if contextually irrelevant (eg, referring to weight loss features). A total of 385 reviews were sampled and 494 segments of text were coded (Multimedia Appendix 5 for full table).

#### Interrater Reliability

The interrater reliability between coders for the general comment theme was initially moderate (kappa=0.51). Following resolution of discrepancies, very high levels of interrater agreement were observed (kappa=0.99).

#### Content

**Figure 3:**
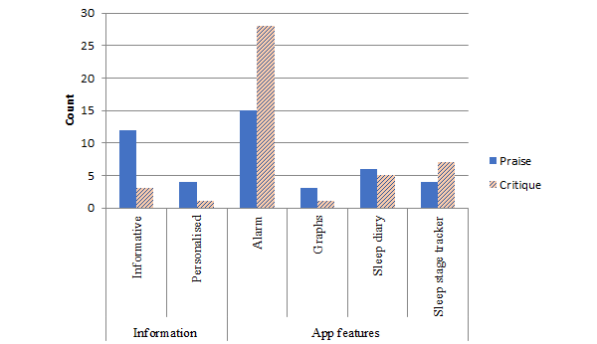
Bar graph presenting frequency of mentions for app content.

Approximately half (66/122, 54%) of *content* mentions were praise for the app. App features were most frequently commented on (100/122, 82%). The alarm was the most salient *app feature* (43/122, 35%), attracting slightly less praise (15/43, 35%) than critique (28/43, 65%). The sleep diary was the next most salient *app feature* with 9% (11/122) of mentions, most of which were praise (6/11, 55%). Informative content was the most commonly mentioned *information* aspect (15/122, 12%) of which most mentions were praise (12/15, 80%; Figure 3).

#### Functionality

**Figure 4:**
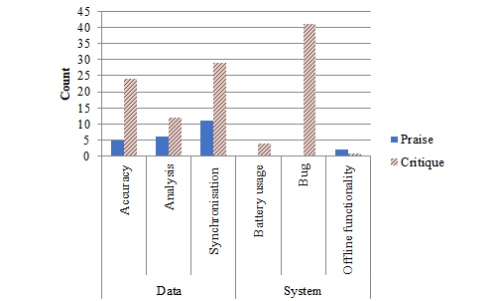
Bar graph presenting frequency of mentions for functionality.

Data synchronization was the most commonly mentioned functionality (40/135, 30%), the majority of which were critiques (29/40, 73%). Bugs were the second most frequently mentioned (41/135, 30%). Mentions of data accuracy followed (29/135, 21%), which included a high proportion of critiques (24/29, 83%; Figure 4).

#### User Experience

Apps’ effectiveness received the highest proportion of *user experience* mentions (29/51, 57%). The general design or esthetics received the second highest proportion of mentions (13/51, 25%).

#### Other

App cost received over half of the *other* mentions (32/53, 60%), which were mostly critiques. In-app purchases were the second most frequently critiqued (11/53, 21%).

## Discussion

### Overview

This study explored the user preferences and needs of individuals with poor sleep or insomnia symptoms to guide the future development of a sleep mobile app. We found most participants expressed interest and willingness to use mobile apps for sleep. The triangulation of results was largely consistent, revealing that sleep app content and functionality are the most important components affecting user experience, which is consistent with previous findings [20,22-24].

### Common Themes/Universal to mHealth Apps

Several generalizable themes emerged while exploring mHealth apps relating to sleep problems. This was in addition to themes common across app development where engagement is maintained by usability, intuitiveness, and bug-free designs [23-26]. Bugs were the most commonly reported problem. App reviewers criticized bugs affecting the app content and functionality. They emphasized the potential utility of these features that was impeded by the bug.

In both our focus groups and app reviews, the value of good quality information related to sleep health was most important. Previous studies have similarly highlighted the significance of relevant informative content that is central and accessible within digital treatments for cystic fibrosis [27] and harmful drinking in young adults [20]. We found that within our focus group sample, the type and extent of information in digital treatments can have counterproductive emotional and motivational consequences. Feelings of condescension from tedious information have also been found in cohorts with eating disorders [28,29] and healthy young adults [30], where content and reminder features are perceived as didactic. These responses may prompt individuals to question continuation and engagement with an app. In light of this, app developers need to ensure educational content is presented in a relevant and meaningful manner and tested with actual end users.

Interestingly, these feelings of belittlement were not echoed by the app reviews. This is perhaps due to contrasting sample characteristics between app reviews from the general public and focus group participants. Our long-term insomnia sufferers in the focus groups were recruited from a clinic and had prior exposure to basic sleep health information delivered in cognitive behavioral therapy for insomnia (CBTI) programs. A recent qualitative study revealed that many insomnia patients believed they knew about and had already attempted sleep hygiene practices [31]. They appeared to switch off when being readvised these from health professionals [31]. Further, given that our focus groups were exploring both app and web resources, the generally repetitive content of Web-based CBTI programs which is used to reinforce learnings may also be perceived as demeaning.

Some focus group participants found existing content to be complex and overwhelming. Resources expected impractical time commitments without a clear and pragmatic rationale for their participation. A delicate balance is necessary between content that is not too basic and perceived as condescending and being too complicated and overwhelming. Dennison et al highlighted how the challenge of burdensome and effortful apps can cause users to disengage [30]. Instead of text-heavy information, the answer may lie in visual and interactive content, conversational interfaces, and offering the user more autonomy, for example, to skip or drill down to more detailed information by choice. This sense of autonomy in users has shown to be important for motivation and engagement [32].

These techniques allow users to personalize content according to their needs which participants highlighted as an important requirement. Personalization also meant different things to different users. Our focus group participants stressed the importance of information personalization; the program adapting to an individual’s symptoms while survey open-ended questions suggested customizability of app features, for example, relaxing sounds and meditation voices were desirable. Given the considerable heterogeneity in preferences, an innovative approach to personalization may involve an initial questionnaire to assess certain design requirements as well as users’ severity and chronicity of sleep disturbance and filter app content accordingly. This customization ensures users are receiving meaningful information that can help sustain engagement without either trivializing or over complicating the user journey.

### Themes Specific to Sleep Apps

Across methods, there were commonly desired app features specific to sleep, for example, alarm clocks. Given that most individuals already utilize the alarm clock in their mobile phones (68%), leveraging this in the design of an app may promote user engagement. Although the alarm is a salient app feature, it attracted more critique than praise in the app reviews, usually due to bugs, suggesting it may detract from a user’s experience and retention when poorly implemented. The focus group participants added that an ideal alarm should go beyond the basic functionality of waking from sleep, involving customizable functionality with creative and sophisticated methods to wake you up and ensure you stay awake. Interestingly, this is in conflict with clinical practice which advises against distractions (including mobile phones) during bedtime [33]. Where major differences exist between user and clinical requirements, further research is required in reaching a compromise between evidence-based aspects and app features that engage.

The second major theme specific to sleep mobile applications was the ability to track sleep quantity and quality. Both focus group and app review participants were interested in synchronization with a WD to track and review their sleep. In addition to the advantages of WD tracking such as objective sleep metrics and sleep stage visualization, WD synchronization may increase usability by reducing burden related to manual sleep data entry. Interestingly, the importance of sleep tracking was echoed by only half of the survey sample. This discrepancy may reflect a selection bias inherent in the app review sample as all selected apps included WD synchronization, compared with only one-third of the survey sample being WD users. Correspondingly, a secondary analysis (not reported in the results) showed that WD users from the survey sample were more likely to indicate the importance of WD synchronization. Of these WD users, sleep tracking was identified as one of the most commonly used WD feature, consistent with previous research [34]. Given the anticipated proliferation in the global health care WD market [35], the addition of WD linkage is vital to the development of sleep-related mobile apps.

In addition to sleep tracking, participants suggested that subjective input supplementary to objective data can help data interpretation. Specifically, both focus group and survey users were interested in recording subjective lifestyle factors tracked against their sleep. In line with our findings, previous studies have shown similar needs for tracking contextual factors that assist in data interpretation to identify potential causes for poor sleep [31,36]. The integration of both subjective and objective sleep data tracking is important in supporting data interpretation and thereby enhancing engagement.

Another feature that may be offered with customization options is the ability to share sleep data with clinicians. Our results showed that those with high insomnia severity (ISI ≥17) were more likely to consider this function valuable in a sleep app. This is consistent with research showing the propensity for health care utilization is greater in those suffering from increased severity of sleep disturbance [37]. However, it is unclear whether clinicians are ready to use WD sleep data in clinical practice owing to a paucity of validation with traditional sleep technologies (eg, polysomnography). Given its potential utility, the guidance of sleep clinicians would be beneficial in determining which sleep data are of clinical value.

### Limitations

Our study has several limitations. First, those who agreed to participate in the focus group and survey were potentially more technology literate and so may not help the development of apps seeking to attract new users. Similarly, app reviewers are likely to be confident technology users given their engagement in a public forum. Despite this probable response bias, the survey sample consisted of a wide age range and varying levels of experience with health apps, providing a good representation of current mobile phone owners who would be potential app users.

Given there was a gender imbalance between the survey sample (88% female) and a male-dominated focus group sample, and we do not know the gender mix of the app reviewers, this may explain some difference between these method’s outcomes. This may reduce the generalizability of our findings.

In addition, we cannot ascertain the extent of sleep disturbance in the app reviewers. Although all apps aimed to improve sleep and only sleep-relevant reviews were analyzed, one app addressed health more broadly and potentially attracted a diverse sample including healthy sleepers.

It is also worth noting that app reviews for previous app versions in the App Store are automatically removed once developers release a new version. Therefore, the study represents a snapshot of app reviews for sleep apps as of February 2018.

### Conclusions

mHealth apps are usually posited at early intervention as either standalone or part of stepped-care programs; however, both require apps with sufficient engagement to enable therapeutic effects. To inform the development of a sleep mobile app, a user-centered approach is adopted to understand the needs and preferences that drive individuals with sleep disturbances to engage with sleep apps. Our findings highlight the importance of app content and functionality. In particular, developers should ensure the personalization of app content, including the customization of app features and information. Informative content should be targeted to the users’ individual sleep experience (the nature and extent of disturbance). Furthermore, developers need to consider including a sleep diary and alarm as a minimum but bear in mind that users seek more sophistication with customizable functionality and sleep diaries with subjective and objective WD tracking. Although further research is required to determine the efficacy of sleep apps, employing user-centric designs to develop engaging apps that are evidence based offers a new opportunity to advance clinical care for sleep.

## Appendix

Multimedia Appendix 1 Online survey.

Multimedia Appendix 2 Full coding scheme.

Multimedia Appendix 3 Main themes and subthemes from workshops.

Multimedia Appendix 4 Characteristics and features of sleep apps in the sample.

Multimedia Appendix 5 Full table of coding frequencies by category.

## Abbreviations

CBTI: cognitive behavioral therapy for insomnia
ISI: Insomnia Severity Index
mHealth: mobile health
PSQI: Pittsburgh Sleep Quality Index
WD: wearable device
