# Supplementary material for: Exploring User Needs and Preferences for Mobile Apps for Sleep Disturbance: Mixed Methods Study
Source: JMIR Ment Health. 2019 May 24;6(5):e13895. doi: 10.2196/13895 (PMC6707571; doi:10.2196/13895)
Supplement: Multimedia Appendix 1 [file mental_v6i5e13895_app1.pdf]

Appendix 1: Online survey

The Sleep App Survey

We're working to create a mobile App to improve people's sleep. This survey will help us understand more about what would be relevant and helpful to the people who would use the App.

1. **What is your gender?**

☐ Male
☐ Female
☐ Prefer not to say

2. **What is your age?**

……… (years)

3. **Which of these best describes the highest level of education you have completed?**

☐ Secondary school
☐ Trade certificate
☐ Diploma
☐ Bachelor degree
☐ Postgraduate Degree

4. **What is your current employment status? (select all that apply)**

☐ Employed full time (40 or more hours per week)
☐ Employed part time (up to 38 hours per week)
☐ Unemployed
☐ Student
☐ Retired
☐ Other (please specify): ………….

If answered employed in question 4, complete question 5

5. **What is your current occupation?**

………………………

About your phone

6. **How often do you use a smartphone?**
   (eg. iPhone, Samsung Galaxy or other touch screen mobile phone)

   ☐ Never ☐ Daily ☐ Weekly ☐ Less often than weekly

7. **What type of phone do you use?**
   (eg. iPhone 5, HTC One, Samsung Galaxy 4, etc.)

Wearable Devices

1. **Do you use a wearable device? (e.g. pedometer, fitness tracker, smart watch etc.)**
   ☐ Yes
   ☐ No

If answered yes to question 1, complete questions 2, 3 and 4.
If answered no to question 1, skip to 'Your Sleep Context'.

2. **Which brand of wearable device do you use? (e.g. Fitbit, Nike, Apple Watch, Garmin)**

   ………………………..

3. **What do you use the wearable device for? (select all that apply)**
   - o Communication
   - o Fitness
   - o Health
   - o Navigation
   - o Sport

4. **What features of the wearable device do you use? (select all that apply)**
   - o Breathing or relaxation exercises
   - o Heart-rate monitoring
   - o Sleep tracking
   - o Step tracking
   - o Stress tracking
   - o Sports or physical activity tracking
   - o Workout intensity
   - o Other (please specify): …..

Your sleep context

1. **How often do you keep your phone by your bed at night?**

   ☐ Almost always ☐ Sometimes ☐ Occasionally ☐ Never

2. **How often do you use your phone as an alarm clock?**

   ☐ Almost always ☐ Sometimes ☐ Occasionally ☐ Never

3. **How often do you use your phone to help you wake up in the morning (eg. by checking email or web browsing as soon as you wake)?**

   ☐ Almost always ☐ Sometimes ☐ Occasionally ☐ Never

The ideal sleep app

1. **If an app were developed that <u>guided you through a program proven to improve sleep</u>, how likely do you think you would be to try it?**

   ☐ Very unlikely ☐ Unlikely ☐ Likely ☐ Very likely

2. **If an app were developed to help you <u>optimise your sleep, in order to improve productivity and performance</u>, how likely do you think you would be to try it?**

   ☐ Very unlikely ☐ Unlikely ☐ Likely ☐ Very likely

3. **If an app were developed to help you <u>discover your true individual sleep needs</u> (how many hours you really need each night), how likely do you think you would be to try it?**

   ☐ Very unlikely ☐ Unlikely ☐ Likely ☐ Very likely

4. **If an ideal sleep app were developed, how important do you think it would be for it to...**

   a. Include a sleep diary and tracking…

   ☐ Unimportant ☐ Slightly important ☐ Important ☐ Essential

   b. Allow tracking of diet, exercise and other lifestyle factors…

   ☐ Unimportant ☐ Slightly important ☐ Important ☐ Essential

   c. Let you share or compare sleep data with friends or other users …

   Unimportant ☐ Slightly important ☐ Important ☐ Essential

   d. Let you share sleep data with your doctor…

   ☐ Unimportant ☐ Slightly important ☐ Important ☐ Essential

   e. Link to a wearable tracking device (eg. Fitbit, Jawbone, ActiWatch)**…**

   ☐ Unimportant ☐ Slightly important ☐ Important ☐ Essential

   f. Be usable offline (without an internet connection)…

   ☐ Unimportant ☐ Slightly important ☐ Important ☐ Essential

   g. Not require the phone to be by the bed…
   ☐ Unimportant ☐ Slightly important ☐ Important ☐ Essential

Apps you have used

1. **Do you use any health apps on your phone? *If yes,*** *please list any that come to mind… (eg. Fitbit, Food diary, MyFitness Pal, etc.)*
2. **Have you ever downloaded any apps for helping you sleep?** They don't necessarily have to be designed for sleep specifically (eg. relaxation music, meditation app, etc.) ***If yes****, please list any that come to mind…*
   a. For any apps you listed above…
   List some things you liked about them:
   List some things you **did not like** about them:
3. **Have you ever used any websites for improving sleep? If yes,** *please list any that come to mind… (eg. Shut-i)*
   a. For any websites you listed above…
   List some things you liked about them:
   List some things you **did not like** about them:

One last thing…

Anything else that would make the perfect

sleep app? What should we include? What should we avoid?

I would like to take part in the prize draw for 1 of 5 Westfield Gift Cards valued at $50.

Yes ☐
No ☐

I am happy to provide the following mobile number and if successful in the Prize Draw be contacted by a member of the research team to receive the prize.

____________________________________Mobile Number

If you would like to receive a summary of our findings following the study, please enter your email address here: ____________________

Great! You have now finished! Thank you for taking the time to complete this survey. Your participation is appreciated.

If you have any questions or concerns regarding this questionnaire please contact the Chief Investigator,
Dr Christopher Gordon
