# Supplementary material for: Exploring User Needs and Preferences for Mobile Apps for Sleep Disturbance: Mixed Methods Study
Source: JMIR Ment Health. 2019 May 24;6(5):e13895. doi: 10.2196/13895 (PMC6707571; doi:10.2196/13895)
Supplement: Multimedia Appendix 2 [file mental_v6i5e13895_app2.pdf]

Appendix 2: Full coding scheme

Category		Description	Example text segment
**General comment**		Brief comment	“I like the app”
**Content**			
**Information**	**Informative**	Referring to the app having informative, interesting or useful information.	“Very nice app with a lot of information”
	**Evidence-based**	Referring to app content that is supported by evidence-based research	“it's based on research CBT-I therapy”
	**Personalised**	Referring to patient-specific content that is tailored according to user input. Includes comments referring to feedback	“The lessons are short and not customized to what the sleep diary discloses”
**App features**			
	**Alarm**	Feature that wakes up the individual during sleep	“I would highly recommend as an alarm or just something to keep track of your sleep.”
	**Sleep diary**	Feature that allows recording of sleep data (e.g. time in bed, time spent sleeping, rising time)	“Sleep tracker is fun to enter daily.”
	**Automatic sleep detection**	Feature that enables the automatic recognition of sleep onset and sleep offset	“If you could update the watch<br><br>app to detect when you go to sleep like many apps already do, this app would be my number one choice!”
	**Sleep stage tracker**	Feature that displays the stages of your sleep using your wearable device data	“Sleep tracking is just ok. It will show me in deep sleep in the morning when I have forgotten to stop it, so it brings the rest of the sleep quality into question”

	**Sound recorder**	Feature that records sound during sleep (e.g. snoring)	"the mic picks up sounds very well"
	**Meditation/relaxation**	Feature that guides you through meditation, gives you instructions to meditate, relaxing sounds or provides you with similar relaxing activities	"Add some features, especially meditations"
	**Notifications**	Feature that reminds or informs you to complete a daily task or check in	"The email notifications on daily tasks was helpful (though a reminder in my calendar could do the same)"
	**Chat-bot**	Feature that simulates a conversation	"I enjoy chatting with the coach, it's like you really have someone that is pushing you to do your best."
	**Goals**	Feature that allows you to set predetermined goals or outcomes	"Lots of good customizable information about my sleep goals"
	**Graphs**	Feature that shows your sleep information in graphical format	"it has real tools (like visualization, charts of your stats"
**User experience**			
**Non-Instrumental**	**General design/Aesthetic**	This is a comment about the design features. It may not differentiate between usability vs aesthetic	"I like the design of the app"<br>"the app is well designed and has a clean aesthetic"<br>"Not helpful. Interface is not intuitive."
**Instrumental**	**Effectiveness**	Refers to the extent and ease to which users can accurately carry out the intended use of the application. This includes comments about the app being easy to use, convenient, useful. Also includes comments referring to the app being too easy to use (criticism)	"App is not intuitive to use to me, despite their video intro."<br>"I love the app but it requires fiddling with your phone when you get in bed in order to track sleep"<br>"Negatives: – … The lessons are short "

			“Having to open both Apps (phone and watch) and hit START, is really lame when you don’t have to on others”<br><br>“Steps to going to sleep are to grab your phone, open the app, click start go to your watch open the app Click start. Then when you wake up in the morning you have to do the same thing in reverse. No thank you”
**Emotional Reactions**	**Motivation**	Refers to the ability of the app to stimulate users interest and drive them to use the app to achieve their goals. Examples include statements about the app being motivating, feeling accountable, encouragement, keeping people focused and on track.	“This app motivates me and it also helps me to not go overboard”<br><br>“Keeps you honest and keeps you engaged”
	**Enjoyment**	Refers to a pleasurable user experience. Examples include statements about enjoying the app and finding it humourous.	“The info provided is interesting and presented in a fun manner”<br><br>“I smile every time it pops up!”
**Functionality**			
**Data-**	**Synchronisation**	Refers to the connection and data transfer of sleep data between:<br><br>- the app being reviewed and wearable device, or;<br><br>- the app being reviewed and a health app (e.g. Healthkit)	“The first night I recorded my sleep data my Apple Watch popped up in the morning saying data analysis failed and to restart my watch”<br><br>“It works especially well with a smart watch, but also

			works well just with a regular device."
	**Accuracy**	Refers to the perception of accuracy/inaccuracy of sleep data	"I have noticed a few times when I've tried to lay down for a nap that it sometimes mistakens that I have slept for a few minutes, even though I know I have not."
	**Analysis**	Refers to the analysis conducted by the app to generate data summaries. This also includes sleep data insights such as time stamps for sleep and sleep metrics	"The app crunches data fast and gives good written summary on progress made and changes to your averages<br><br>"I'd really love to see the app updated to establish averages and daily activity on device"<br><br>"It's also disappointing to see a lack of granularity in my sleep data."
**System**	**Bug**	Refers to an error in the app that produces an unintended outcome	"It force quits every time I open it."
	**Offline functionality**	Refers to app features that work offline	"I like this app, but I hate that I can't update it without wifi."
	**Battery Usage**	Refers to phone or wearable device battery usage by the app	"Excessive battery usage on a Pixel 2"
**Other**			
	**Trial Period**	Refers to the trial period provided before purchasing the app	"A trial period before committing $300 upfront payment would be more enticing even for this sleep deprived exhausted desperately seeking solutions noodle."

	**Judgmental**	Refers to perceiving the app as making judgmental or non-judgmental comments. This may include comments about feeling condescended or belittled	"It feels like gentle encouragement without judgement."<br><br>"I need something to help guide me rather than condemn the choices I make."<br><br>"I also appreciate the way it gives you honest feedback without being negative."
	**Privacy**	Refers to the collection of data by the app that poses privacy concerns	"More data mining by "free" app"<br><br>"the thought of recording your dream in a third party database accessible from the web is a little creepy"
	**Ads**	Refers to in-app advertisements	"The ads for upgrading to the premium (paid) version are so obnoxious it makes the free<br><br>copy unusable"
	**App Cost**	Refers to the cost of an app	"It would be really nice if we could access premium features without paying. I think we shouldn't have to pay to know about our sleep."
	**In-app Purchases**	Refers to additional features purchased by the user	"I have bought the add on feature which involves a smart alarm and I like it"
