# Supplementary material for: Exploring User Needs and Preferences for Mobile Apps for Sleep Disturbance: Mixed Methods Study
Source: JMIR Ment Health. 2019 May 24;6(5):e13895. doi: 10.2196/13895 (PMC6707571; doi:10.2196/13895)
Supplement: Multimedia Appendix 3 [file mental_v6i5e13895_app3.pdf]

Appendix 3: Main themes and subthemes from workshops

**Theme**	Description	**Subtheme**	Description
**App content**			
**Information**	Characteristics of an ideal sleep app.	**Adaptive sleep program**	An app that adapts recommendations and the structure of its program to the behavior and circumstances of the user.
		**Multiplatform**	Usable on larger screens (ie. desktop computers) as well as mobiles or tablets.
		**Lighthearted friendly tone**	Participants favoured a fun lighthearted approach over an overly serious approach that makes the program feel like work.
		**Avoid complex text-heavy content**	Several participants expressed frustration at existing online content and exercises for sleep being “too complicated”, “overwhelming”, “tedious” and “long-winded”.
		**Avoid Condescending tone of existing resources**	Some participants reported that some sleep programs or online content felt “demeaning” or “condescending” or made them feel like children, for example by presenting overly basic content and quizzes.
		**Cost as motivator**	On participant expressed frustration at her experience with another sleep program (Shut-i) pointing out that the only reason she completed it was that she’d paid $200 for it.
		**Culturally tailored**	Participants expressed a preference for content in their own accent and culture.
		**Clear rationale and feedback**	Participants expressed frustration with lengthy surveys without clear rationale or any immediate feedback provided on data collected.
**App features**	Feature preferences for an ideal sleep app	**Alarm**	Participants described alarms to wake you up in the morning but also to keep you awake at night until the program’s prescribed bedtime.
		**Audio features**	Many participants had successfully used some form of relaxation, meditation or podcast audio to assist with sleep and recommended inclusion (either of original content or links to existing content).
		**Engagement via feedback and encouragement**	Participants described various types of encouragement including rewards and badges, displays of progress, encouraging messages and tips and the use of a journey metaphor.
		**Games**	Some participants expressed interest in games, and confirmed interest in an “alertness game” to track daytime alertness throughout a sleep program.
		**Graphics and visuals**	There was an interest in graphs to visualize prograss and data, and the use of a dark interface to avoid exposure to bright and blue light at night.
		**Information and help**	Participants were interested in FAQs and preferred them over a chatbot since less typing into a phone was involved.

		**Recommend-ations**	Participants were interested in recommendations for alarms, apps, podcasts, websites, and sleep research.
		**Sleep tracking and data visualisation**	Participants pointed to the need for tracking a subjective sleep measure such as Sleep “quality”or “Satisfaction” explaining that measures like “hours” can be misleading. There was also interest in the ability to track daily behaviors (ie. exercise, sleeping pill) for example via tags in order to link to sleep.
		**Social features**	Participants expressed interest in ways of sharing experiences with other poor sleepers such as via a user community forum. They were not interested in sharing with friends more generally, for example, via Facebook, explaining that people without sleep problems don’t understand or sympathise with the experience. They also confirmed interest in the option of nominating a support person.
		**Staying awake**	Ideas for help staying awake (for example as part of Sleep Restriction Therapy) included the app providing personalized stimulating content (eg. News, sport), wakeful games and a motion-detecting alarm that could go off with lack of motion.
**Sleep experiences**			
	Lived experience of difficulty with sleeping including sleep strategies.	**Bad for sleep**	Participants reported the following as being bad for their sleep: Screen light, a disorganized bedroom, podcasts without timers to turn them off automatically, devices near the bed, social media and entertainment apps, and partner snoring.
		**Lived experiences of poor sleep**	Participants shared difficulties arising from frequent night wakings, trouble falling asleep, nightmares, nasal congestion and snoring, bothering partner, mind racing, daytime stress, daytime sleepiness, anxiety over not sleeping, lack of any sleep rhythm and lack of sympathy from normal sleepers.
		**Sleep strategies**	Sleep strategies of participants included keeping a dark room, reading, using relaxation techniques (ie. relaxing sounds, mindfulness), watching television, use of white noise, ear plugs, podcasts for sleep, keeping feet warm, and online sleep content.
