# Supplementary material for: Exploring User Needs and Preferences for Mobile Apps for Sleep Disturbance: Mixed Methods Study
Source: JMIR Ment Health. 2019 May 24;6(5):e13895. doi: 10.2196/13895 (PMC6707571; doi:10.2196/13895)
Supplement: Multimedia Appendix 4 [file mental_v6i5e13895_app4.pdf]

Appendix 4. Characteristics and features of sleep apps in the sample.

The mobile apps were manually coded for content (MA) and developed inductively based on common sleep app features. Each app was downloaded onto an iPhone 6 and Samsung Galaxy S7 and each category coded as present or not. Mobile apps with free and premium versions were coded as two separate apps.

	**Sleeprate**		**Pillow**		**Sleepio**	**Sleep as Android**	**SleepHealth**	**Lark**	
**Feature included**	**Free**	**Paid**	**Free**	**Paid**	**Paid**	**Free**	**Free**	**Free**	**Paid**
**Alarm**	N	Y	Y	Y	N	Y	N	N	N
**Sleep diary**	Y	Y	Y	Y	Y	Y	Y	Y	Y
**Graphical Feedback**	Y	Y	Y	Y	Y	Y	Y	Y	Y
**Sound recorder**	Y	Y	Y	Y	N	Y	N	N	N
**Sleep stage data/parameters**	Y	Y	Y	Y	N	Y	N	N	Y
**Goal setting**	N	Y	Y	Y	Y	Y	N	N	N
**Personalised advice based on sleep data**	Y	Y	Y	Y	Y	Y	Y	Y	Y
**Relaxation sounds**	N	N	N	Y	Y	Y	N	N	N
**Alertness checker**	N	N	N	N	N	N	Y	N	N
**Breathing exercise**	Y	Y	N	N	Y	N	N	N	N
**Accelerometer-derived sleep tracking**	Y	Y	Y	Y	Y	Y	N	Y	Y
**Cost**	F	P	F	P	P	F-P	F	F	P
**Notifications/reminders**	N	Y	N	Y	Y	Y	Y	Y	Y
**Sleep hygiene**	N	Y	N	Y	Y	Y	N	N	N
**Gamification**	N	Y	N	N	Y	N	N	N	N

Y, yes; N, no; F, free; F-P, free with in-app purchases; P, paid
