# Supplementary material for: Exploring User Needs and Preferences for Mobile Apps for Sleep Disturbance: Mixed Methods Study
Source: JMIR Ment Health. 2019 May 24;6(5):e13895. doi: 10.2196/13895 (PMC6707571; doi:10.2196/13895)
Supplement: Multimedia Appendix 5 [file mental_v6i5e13895_app5.pdf]

Appendix 5: Full table of coding frequencies by category (n=494)

Category		Praise frequency, n (%)	Critique frequency, n (%)
**General comment**		118 (88)	15 (11)
**CONTENT Category**		66 (54)	56 (46)
**INFORMATION**		18 (15)	4 (3)
	Evidence-based	2 (2)	0 (0)
	Informative	12 (10)	3 (2)
	Personalised	4 (3)	1 (1)
**APP FEATURES**		48 (39)	52 (43)
	Alarm	15 (12)	28 (23)
	Automatic sleep detection	1 (1)	6 (5)
	Chat-bot	8 (7)	0 (0)
	Goals	1 (1)	0 (0)
	Graphs	3 (2)	1 (1)
	Meditation/relaxation	4 (3)	1 (1)
	Notifications	1 (1)	0 (0)
	Sleep diary	6 (5)	5 (4)
	Sleep stage tracker	4 (3)	7 (6)
	Sound recorder	5 (4)	4 (3)
**USER EXP Category**		25 (49)	26 (51)
	Instrumental: Effectiveness	12 (24)	17 (34)
	Non-Instrumental: General design/Aesthetic	5 (10)	8 (16)
**EMOTIONAL REACTIONS**		8 (16)	1 (2)
	Enjoyment	3 (6)	1 (2)
	Motivation	5 (10)	0 (0)
**FUNCTIONALITY Category**		24 (18)	111 (83)
**Data**		22 (16)	65 (49)
	Accuracy	5 (4)	24 (18)
	Analysis	6 (4)	12 (9)
	Synchronisation	11 (8)	29 (22)
**SYSTEM**		2 (1)	46 (34)
	Battery Usage	0 (0)	4 (3)
	Bug	0 (0)	41 (31)
	Offline functionality	2 (1)	1 (1)
**OTHER Category**		6 (11)	47 (89)
	Ads	0 (0)	1 (2)
	App Cost	3 (6)	29 (55)
	In-app Purchases	0 (0)	11 (21)
	Judgmental	1 (2)	2 (4)
	Privacy	0 (0)	2 (4)
